# Predicting Professional Quality of Life and Life Satisfaction in Spanish Nurses: A Cross-Sectional Study

**DOI:** 10.3390/ijerph17124366

**Published:** 2020-06-18

**Authors:** Noemí Sansó, Laura Galiana, Amparo Oliver, Macià Tomás-Salvá, Gabriel Vidal-Blanco

**Affiliations:** 1 Department of Nursing and Physiotherapy, University of the Balearic Islands, Spain and Balearic Islands Health Research Institute (IDISBA), 07122 Palma de Mallorca, Spain; Noemi.Sanso@uib.es; 2 Department of Methodology for the Behavioral Sciences, University of Valencia, 46010 Valencia, Spain; oliver@uv.es; 3 Prevention of Occupational Hazards Service, Government of the Balearic Islands, 07010 Palma de Mallorca, Spain; mtomas@dgfun.caib.es; 4 Department of Nursing, University of Valencia, 46010 Valencia, Spain; Gabriel.Vidal@uv.es

**Keywords:** self-care, occupational health, burnout, quality of life, nursing

## Abstract

*Background*: Dealing with suffering, grief, and death on a daily basis, together with the particular working conditions, may produce high levels of burnout in nurses, and hinder their well-being. The purpose of this research is to study the effect of self-care and self-compassion on nurses’ professional quality of life and well-being. *Methods*: The research had a cross-sectional design, used correlational methodology and a structural equation model was hypothesized. Along the study, 210 nurses from the Healthcare Public System of the Balearic Islands, participated. The study took place from June to September 2018. *Results*: The hypothesized model showed an overall adequate fit. Practice environment predicted both self-care and self-compassion, whereas nursing stress did not. Self-care and self-compassion predicted nurses’ professional quality of life, whereas the practice environment and nursing stress were not predictors. Finally, professional quality of life showed a positive relationship with life satisfaction. *Conclusions*: The study presents a comprehensive structural equation model in which self-care and self-compassion are the best predictors of nurses’ professional quality of life. A direct relation of professional quality of life and nurses’ well-being has also been found, while controlling for the effects of nurses’ practice environment and stress.

## 1. Introduction

Nursing has been traditionally identified as a very stressful profession, ‘by its very nature’ [1]. There is a vast body of research studying stress in nursing that has pointed how dealing with suffering, grief, and death on a daily basis, together with the particular working conditions may produce high levels of occupational stress and burnout and, therefore, may hinder nurses’ well-being [2,3].

A continued exposition to stress can lead to the development of burnout: a psychological state resulting from a long-lasting psychological or emotional stress [4], characterized by emotional exhaustion, depersonalization, and low personal accomplishment [5]. Several negative consequences of burnout have been described in the context of healthcare professionals, such as insomnia, irritability and alcohol and drug use [6] (pp. 245–264). Adverse effects have been also identified for the healthcare system, including absenteeism, sick leaves, suboptimal care of patients, or treatment errors, consequently affecting quality of care [7].

In this arena of study, more and more research has pointed that occupational stress and BO for themselves are insufficient for a comprehensive approach to professional quality of life [8,9]. As defined by Satmm [9], professional quality of life is “the quality one feels in relation to their work as a helper” (p.8). This professional quality of life would include both the positive and negative issues of doing one’s job, which are, according to this model: BO, compassion fatigue (CF), and compassion satisfaction (CS) [9]. CF has been defined as the negative effects derived from caring traumatized individuals [10], provoking “debilitating weariness brought about by repetitive, empathic responses to the pain and suffering of others” [11]. A continued exposition to patients’ suffering produce a chronic tension and preoccupation, with negative effects such as psychological difficulties, emotional and physical exhaustion, inability for compassion, or a diminished resistance to others’ suffering [12] (pp. 9–23). CF symptoms include apathy, depression, errors in clinical judgement, sleep disorders, hypertension, feeling of impotence, irritation and emotional suppression, anxiety, and even poor quality of work [13].

Specifically in nurses, CF has been defined as “the final result of a progressive and cumulative process that is caused by prolonged, continuous, and intense contact with patients, the use of self, and exposure to stress […] is a state where the compassionate energy that is expended by nurses has surpassed their restorative processes, with recovery power being lost” [14]. Compassion fatigued nurses would exhibit apathy, depression, errors in clinical judgement, sleep disorders, hypertension, feeling of impotence, irritation and emotional suppression, and even poor quality of work, being determinant in patient safety and quality of care and compromising a compassionate care [13,15]. High levels of compassion fatigue have recently been reported in several nursing specialties, such as emergency nurses [16,17], heart and vascular nurses [18], oncology nurses [19,20], midwives [21], critical care nurses [22], or pediatric nurses [23], and in samples from different countries, including Australia [24,25], Canada [26], Ireland [27], South Africa [28], the UK [29], or the US [30,31,32].

However, working with those who suffering does not only have negative consequences, but rather can result in satisfaction for the professional [33]. When nurses adequately address stress and suffering, they can receive pleasure and satisfaction when helping others, instead of compassion fatigue and burnout syndrome. This phenomenon has been called compassion satisfaction [9], and has been defined as exquisite empathy or compassion [34]. As aforementioned, CS, together with BO and CF, have been conceptualized as the three dimensions of professional quality of life when studied in the context of the healing relationship [9].

Among professional quality of life determinants, self-care and self-compassion have been pointed as essential for providing compassionate care and maintaining nurses balance [35]. Self-care has been conceptualized as a cadre of activities performed independently by individuals to prevent illness and maintain and promote personal well-being throughout life [36]. Accomplishing self-care activities plays an important role in helping professionals to cope with the emotional demands they have to face every day, being vital for both nurses and patients [35]. Indeed, a holistic practice of self-care is a key aspect for maintaining health and professional quality of life [37]. For example, Neville and Cole [38] and in a sample of nurses practicing in a community medical center, found evidence of a negative relation between self-care and compassion fatigue. More recently, Sorenson [39] found, after a review of qualitative articles, that self-care was reported to be the most significant preventative measure healthcare professionals could take to protect themselves from developing compassion fatigue.

Self-compassion, in turn, has been defined as compassion directed towards oneself, extending compassion to ourselves as we would to others [40]. Self-compassion allows the healthcare professional to build resilience against stress and burnout [41]. Recent research has found an association between self-compassion and nurses’ professional quality of life. For instance, Gustin and Wagner [42] discovered that cultivating compassion in nursing professionals improved compassion for others. Durkin et al. [43] found that more self-compassionate nurses were less likely to experience burnout. Along the same lines, in the study carried out by Mahon [44], nurses’ perceived stress decreased after an intervention based on mindfulness and self-compassion, whereas levels of compassion showed a statistically significant increase. Additionally, self-compassion allows better interpersonal work and is related to other professional quality of life determinants, such as empathy [45,46].

The aim of this study is to study the effect of self-care and self-compassion on nurses’ professional quality of life and well-being, specifically, on life satisfaction. Life satisfaction has been defined as a cognitive, judgmental process [47], in which person’s quality of life is globally assessed according to his/her chosen criteria [48]. Thus, it is a conscious cognitive judgment, based on the comparison of one’s life with a self-imposed standard or set of standards, which lead to a global assessment of life [49]. For this purpose, we tested a structural equation model in which self-care and self-compassion predicts nurses’ professional quality of life when controlling for the traditional variables related to this construct (i.e., nurses’ stress and nursing work environment). Additionally, the relationship between nurses’ professional quality of life and their well-being is tested. Taking into account the aforementioned literature, our hypotheses are the following:

**Hypothesis 1** **(H1).** *Nurses’ stress and nursing work environment will affect their levels of self-care and self-compassion and professional quality of life. Specifically, stress will be negatively related to lower levels of self-care, self-compassion, and professional quality of life (H1A), whereas nursing conditions will be positively related to these variables (H1B).*

**Hypothesis 2** **(H2).** *Nurses with higher levels of self-care and self-compassion will present higher levels of professional quality of life. That is, both self-care and self-compassion will be positively related to professional quality of life.*

**Hypothesis 3** **(H3).** *Nurses with higher levels of professional quality of life will present higher levels of well-being. That is, there will be a positive relationship between nurses’ professional quality of life and life satisfaction*.

## 2. Materials and Methods

### 2.1. Design, Procedure, and Participants

The study was carried out through a longitudinal panel design, with measurements from a sample of nursing professionals from the Balearic Islands (Spain) at three-time points. Data from the first wave were used. We employed correlational methodology, as the correlational method involves looking for relationships among variables. In our case, the aim was to study the relations among nurses’ stress, nursing work environment, self-care, self-compassion, nurses’ professional quality of life, and well-being

First of all, nursing managers of the health centers of the Balearic Islands (specifically there were 14 centers, with a total of approximately 4336 employed nurses) were invited to participated, first with a written letter, and after with personal interviews in which the research project was explained in detail. Once the permission was obtained, each nursing manager was asked to send the invitation letter to the nurses of the center. This invitation was send by mail, with the link to the survey, which was hosted in the online platform SurveyMonkey^®^. In this same platform participating nurses signed the informed consent. Data confidentiality was ensured. The study took place from June to September 2018.

Participants were nurses working in the Healthcare Public System of the Balearic Islands at the moment of the study. Those nurses not working in the moment of the survey or working exclusively in administration tasks (not developing care activity) were excluded in order to address potential sources of bias. A minimum sample size of 200 was established following Kline’s [50] recommendation. Eventually, 210 nurses participated (4.8%).

### 2.2. Instruments

The survey included the following scales:

- The Short version of the Nursing Stress Scale (Short NSS). The Nursing Stress Scale (NSS) was designed to hospital nurses in 1981 [51] and was validated into Spanish in 1999 [52]. It assesses six dimensions of nurses’ occupational stress with 34 items: coping with death and dying process, conflict with physicians, lack of staff support, conflict with other nurses, workload, uncertainty concerning treatment, and work role setting. The short version uses one indicator per dimension, and respondents scores range from 0 (never) to 3 (always). In this study the research team have used a short version and the reliability of this scale in this sample was 0.701.
- The Short version of the Practice Environment Scale of the Nursing Work Index (PES-NWI). The Practice Environment Scale of the Nursing Work Index (PES-NWI) was developed by Lake [53] and, ten years later, it was validated into Spanish [54]. The scale evaluates five dimensions of nursing work environment with 31 items: nurse participation in hospital affairs; nursing foundations for quality of care; nurse manager ability, leadership and support for nurses; staffing and resource adequacy; collegial nurse–physician relations. The short version uses one indicator per dimension, and respondents scores range from 1 (totally disagree) to 4 (totally agree). The internal consistency estimate of the short version used in the study was 0.740.
- The Professional Self-Care Scale (PSCS) [37]. The original Spanish version was used. It is composed by nine items and assesses three dimensions of professionals’ self-care: physical, which refers to the implication in activities that helps to maintain a healthy body; inner, which is related to activities that help to keep a healthy mind; and social, regarded to activities related to social activities that help the individual to maintain social health. Items score in a 5-point Likert-type scale, from 1 (totally disagree) to 5 (totally agree). The reliability of the scale in this sample was 0.730.
- The Self-Compassion Scale (SCS) [55]. In this case, the short version validated into Spanish in 2014 [56] was used. The SCS is formed by 12 items assessing three main components of self-compassion and their opposites: self-kindness/self-judgment, common humanity/isolation, and mindfulness/over-identification. Items score in a 5-point Likert-type scale, from 1 (totally disagree) to 5 (totally agree). The Cronbach’s alpha in this sample was 0.848.
- The Professional Quality of Life Scale (ProQoL) [9]. The ProQOL has been recently validated in Spanish [57]. This scale evaluates three dimensions of quality of life: compassion satisfaction, compassion fatigue, and burnout syndrome, with ten items per factor. In current research, a short version of three items per factor was used. Items score in a 6-point Likert-type scale, from 0 (never) to 5 (always). Internal consistency estimates were 0.872, 0.812, and 0.646. It has to be borne in mind that quality of life was modeled as a latent factor and, consequently, estimated free of error for the prediction purpose.
- Satisfaction with Life Scale [47]. The Spanish version was used [58]. The scale assesses subjective well-being, specifically global satisfaction with life, with five items. Items score in a 5-point Likert-type scale, from 1 (totally disagree) to 5 (totally agree). The internal consistency in this sample was 0.911.

### 2.3. Ethical Considerations

The Ethical Research Committee of the University of the Balearic Islands approved the project (code 82CER18). Throughout the study, the research team has ensured at all times the compliance with the ethical principles of research in health sciences established nationally and internationally: Helsinki Declaration, The Declaration of Geneva of the World Medical Association, and the International Code of Medical Ethics, and the latest APA Ethical Principles of Psychologists and Code of Conduct.

### 2.4. Data Analyses

First, demographic characteristics of the sample, together with descriptive statistics of the variables under study were calculated.

**Figure 1:**
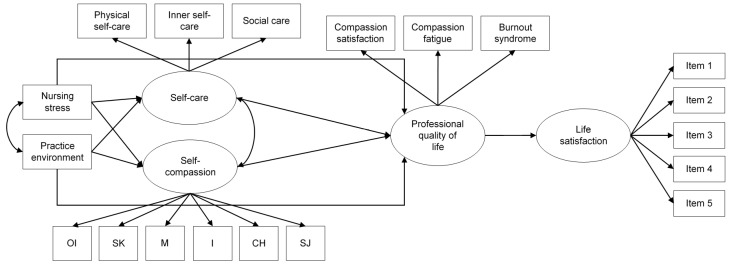
Hypothesized model. OI = over identification; SK = self-kindness; M = mindfulness; I = isolation; CH = common humanity; SJ = self-judgement.

Then, a full structural equation model (SEM) for the prediction of nurses’ professional quality of life and well-being was hypothesized, estimated, and tested. The model hypothesized effects of nurses’ organizational conditions (stress and practice environment, which were correlated) on personal variables (self-care and self-compassion, which were also correlated). These four variables predicted professional quality of life, with self-care and self-compassion having direct effects on nurses’ professional quality of life, and nurses’ stress and practice environment having both direct and indirect effects (partial mediation). Finally, the model also included a direct effect of nurses’ professional quality of life on their well-being, as measured by the five items of the Satisfaction With Life Scale (see Figure 1).

The overall fit of the model was assessed using several fit criteria: the chi-square statistic (*χ*^2^), the Comparative Fit Index (CFI), the Tucker–Lewis Index (TLI), the Root Mean Square Error of Approximation (RMSEA), and the Standardized Root Mean Square Residual (SRMR). Values over 0.90, and ideally over 0.95, for CFI and TLI, and values lower than 0.08 are generally considered a good fit [59]. Additionally, analytical fit was also examined in order to test our hypotheses and evidence was compared with previous literature.

The model was estimated using the mean- and variance-adjusted weighted least squares estimator, with Mplus version 8 software [60]. Missing data were estimated using with Full Information Maximum Likelihood, which involves less bias and is generally more efficient compared to other missing data techniques [61].

## 3. Results

As regards the demographic characteristics of the sample, mean age was 40.24 years (*SD* = 9.78). 84.5% were women and 11.9% were supervisors. As regards rotation, 52.4% had a fixed turn, whereas 47.6% rotated. Demographic and work conditions are presented in Table 1.

Descriptive statistics of the variables, together with number of participants with missing data for each variable of interest are presented in Table 2. As shown in the table, levels of nursing stress were medium and levels of practice environment were medium, indicating moderate levels of practice environment. As regards self-care, levels were higher for social self-care, with medium levels of physical self-care and inner self-care. As regards self-compassion, levels of the positive subscales were adequate, around the middle point of the scale. However, levels of negative self-compassion were also medium, almost around the middle point. As regards professional quality of life, levels of CS were high, levels of CF were low, and levels of burnout were medium (6.43). Finally, levels in the items of life satisfaction ranged from 2.90 to 3.88, and the mean was 3.30; thus, we could say life satisfaction level was medium-high.

Table 3 includes the levels of the different variables under study, for each demographic and work condition group.

**Figure 2:**
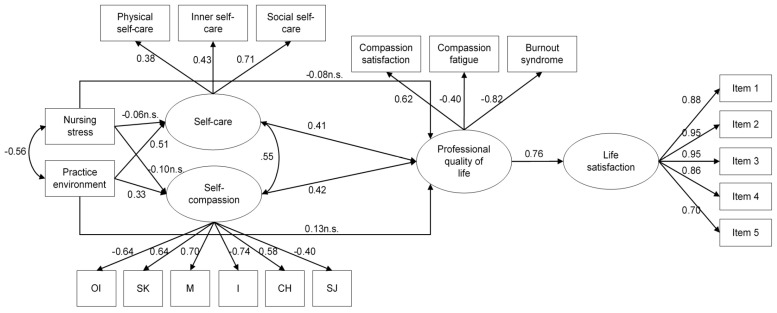
Estimated model. OI = over identification; SK = self-kindness; M = mindfulness; I = isolation; CH = common humanity; SJ = self-judgement. All the relations were statistically significant (*p* < 0.01), except for the ones marked n.s. (non-significant).

Finally, and as regards the model results, the hypothesized model showed an overall adequate fit: *χ*^2^(143) = 298.953 (*p* < 0.001), CFI = 0.942, TLI = 0.931, SRMR = 0.071, RMSEA = 0.080 [90% confidence interval = 0.067,0.093]. As regards the analytical fit, and as it is shown in Figure 2, practice environment predicted both self-care (*R*^2^ = 0.291) and self-compassion (*R*^2^ = 0.155), whereas nursing stress did not. Self-care and self-compassion, in turn, predicted nurses’ professional quality of life (*R*^2^ = 0.734), whereas practice environment and nursing stress were not statistically significant predictors. Finally, professional quality of life showed a positive, high, and statistically significant relation with life satisfaction (*R*^2^ = 0.581). It is worth to note more than two quarters of nurses’ professional quality of life variance was explained by self-care and self-compassion, and more than half the variance of nurses’ life satisfaction by their levels of professional quality of life.

## 4. Discussion

The aim of the study was to test a structural equation model in which self-care and self-compassion predicted nurses’ professional quality of life, when controlling for nurses’ stress and nursing work environment; including, at the same time, a predictive paper of nurses’ professional quality of life on their well-being. The literature-based hypotheses contemplated in the model will guide the discussion of our results.

The first hypothesis, “Nurses’ stress and nursing work environment will affect their levels of self-care and self-compassion and professional quality of life”, was partially supported by the data. Whereas positive relations between nursing work conditions and self-care and self-compassion were found, nurses’ stress was not related to these variables. This aspect has been pointed out in different studies on stress that emphasized the impact of the nurses’ working conditions on their professional quality of life [2,3]. Additionally, H1 also specified direct relationships between professional quality of life and nurses’ stress and working conditions that were not statistically significant in the model. These results are somehow counterintuitive because previous results pointed to stress and work conditions as predictors of quality of life [62]. Aiken et al. [62], for instance, carried out a study to determine whether good organization of care in hospitals (such as improved nurse staffing and work environments) affected patient care and nurse workforce stability in US and European countries, and they found a relationship between the work environment and nurses’ burnout. However, no study has tested the effect of stress and work conditions, controlling for personal variables such as self-care or self-compassion.

Our second hypothesis, “Nurses with higher levels of self-care and self-compassion will present higher levels of professional quality of life”, was fully supported by the data. Positive and statistically significant relationships were found between these variables. These results completely coincide with the authors’ previous studies, carried out in Spanish and Brazilian palliative care contexts [37,63], and they highlight the relevance of both variables in nurses’ professional quality of life.

Taking the results for both H1 and H2 together, we could interpret them in terms of the importance of self-care and self-compassion in preventing compassion fatigue in healthcare professionals. In other words, self-care and self-compassion seem to be the key to predicting professional quality of life, above and beyond organizational factors such as stress or working conditions. As a result of its relation with health professionals’ well-being and the quality of care [7,64], the need of a better comprehension of those determinants of healthcare providers’ professional quality of life has been emphasized [63,65,66]. This research adds evidence in this scientific arena, pointing out the outstanding role of both self-care and self-compassion.

Finally, our third hypothesis, “Nurses with higher levels of professional quality of life will present higher levels of well-being”, was also supported by the data, with professional quality of life being a significant predictor of nurses’ life satisfaction. These two variables were related in the expected direction and fully coincide with Lizano’s [67] review in human service workers showing the detrimental impact of job burnout on the affective, psychological, physiological, and behavioral well-being of workers. This is of special importance, as stress can have a devastating impact, compromising clinicians’ level of performance and the success of interventions, their professional quality of life and job satisfaction, and even their personal well-being [1], as it has been supported by our results. These results, then, provide a better understanding of these professionals’ quality of life, which will contribute to increase motivation and well-being of professionals, but it also will imply lower levels of medical errors, sick leaves and absenteeism [68], and better quality of care [7].

### Limitations

The main limitation of the study is the difficulty to establish relationships of causality in the absence of a longitudinal design. Future research should provide evidence in this line. In addition, there may be limitations to generalize to other populations aside from the Spanish one. The fact that results are limited to a reduced Spanish sample may hinder their generalizability. However, the nature of the variables under study, related to the nurse profession and human nature, together with the use of well-known widely validated measures, make us think similar results will be found in other samples. The manuscript has, however, an important strength, being the first time that both inner variables, that is self-care and self-compassion, together with organizational-related ones, such as practice environment and stress, have been tested to predict not only nurses’ professional quality of life but also they well-being.

## 5. Conclusions

All in all, it can be concluded that the main variables that have been shown to affect nurses’ quality of professional life are those related to personal characteristics instead of organizational ones. Indeed, our results point that nurses’ care and compassion for themselves are the clue for maintaining adequate levels of professional quality of life and, consequently, guarantee nurses’ well-being and patients’ quality of care. Therefore, practical implications of current research include, among others, the implementation of interventions to increase personal variables such as self-care and self-compassion, so that to promote nurses’ professional quality of life and well-being. Taking into account that professional quality of life of healthcare workers is in increased risk with the COVID-19 pandemic crisis, these interventions should be a must for healthcare institutions. Spanish nurses have been committed to our well-being long before this pandemic; they deserve our commitment in their well-being, too.

## Figures and Tables

**Table 1:** Demographic characteristics of the sample.

**Variables**		**M (SD)**
Age		40.24 (9.78)
Years in nursing		3.75 (2.05)
Years in current area/specialty		2.40 (1.74)
Years in current job position		1.86 (1.49)
**Variables**	**Categories**	**N (%)**
Gender	Women	158 (75.2)
	Men	29 (13.8)
	Missing data	23 (11.0)
Shifts	Without shifts	97 (46.2)
	With shifts	88 (41.9)
	Missing data	25 (11.9)
Working day duration	8 h	153 (72.9)
	10 h	5 (2.4)
	12 h	24 (11.4)
	Missing data	28 (13.3)
Job situation	Public worker	119 (56.7)
	Acting official	29 (13.8)
	Temporary worker	39 (18.6)
	Missing data	23 (11.0)

Notes: M = Mean; SD = Standard deviation.

**Table 2:** Descriptive statistics and number of participants with missing data for the variables under study.

Variable	M	SD	Min.	Max.	S	K
Nursing stress (scale 0–3)	1.34	0.48	0.33	2.67	0.39	−0.41
Practice environment (scale 1–4)	2.68	0.53	1.17	4.00	−0.28	−0.09
Physical self-care (scale 1–5)	3.63	0.86	1.33	5.00	−0.36	−0.68
Inner self-care (scale 1–5)	2.61	0.93	1.00	5.00	0.47	−0.35
Social self-care (scale 1–5)	3.93	0.64	2.00	5.00	−0.74	0.66
Over-identification (scale 1–5)	3.01	1.01	1.00	5.00	−0.02	−0.75
Self-kindness (scale 1–5)	3.24	0.83	1.50	5.00	−0.06	−0.35
Mindfulness (scale 1–5)	3.58	0.84	1.00	5.00	−0.33	−0.19
Isolation (scale 1–5)	2.70	1.02	1.00	5.00	0.17	−0.57
Common humanity (scale 1–5)	3.22	0.84	1.00	5.00	−0.17	−0.18
Self-judgement (scale 1–5)	2.81	1.00	1.00	5.00	−0.01	−0.60
Compassion satisfaction (scale 0–15)	12.02	2.82	3.00	15.00	−1.22	1.28
Compassion fatigue (scale 0–15)	3.49	2.95	0.00	11.00	0.72	−0.40
Burnout (scale 0–15)	6.43	3.79	0.00	15.00	0.35	−0.58
Life satisfaction—item 1 (scale 1–5)	2.90	1.14	1	5	0.14	−0.77
Life satisfaction—item 2 (scale 1–5)	3.54	1.01	1	5	−0.58	−0.30
Life satisfaction—item 3 (scale 1–5)	3.10	0.97	1	5	−0.01	−0.32
Life satisfaction—item 4 (scale 1–5)	3.11	1.00	1	5	−0.10	−0.54
Life satisfaction—item 5 (scale 1–5)	3.88	1.05	1	5	−0.76	−0.12
Life satisfaction (scale 1–5)	3.30	0.89	1	5	−0.17	−0.49

Notes: M = Mean; SD = Standard deviation; Min = Minimum score; Max = Maximum score; S = Skewness; K = Kurtosis.

**Table 3:** Means and standard deviations of the variables under the study for demographic and work conditions groups.

Variable	Gender		Shifts		Working Day Duration			Job Situation		
	Women	Men	Without	With	8 h	10 h	12 h	Public Worker	Acting Official	Temporary Working
Nursing stress (scale 0–3)	1.36(0.47)	1.25(0.53)	1.23(0.46)	1.47(0.48)	1.33(0.47)	1.33(0.62)	1.51(0.53)	1.36(0.50)	1.32(0.43)	1.30(0.45)
Practice environment (scale 1–4)	2.70(0.53)	2.59(0.55)	2.85(0.43)	2.50(0.59)	2.69(0.53)	2.71(0.82)	2.60(0.58)	2.68(0.56)	2.63(0.44)	2.74(0.55)
Physical self-care (scale 1–5)	3.62(0.87)	3.70(0.84)	3.79(0.90)	3.47(0.78)	3.64(0.87)	3.92(0.83)	3.45(0.84)	3.68(0.87)	3.82(0.85)	3.35(0.82)
Inner self-care (scale 1–5)	2.58(0.90)	2.76(1.09)	2.69(0.93)	2.55(0.92)	2.64(0.90)	3.13(1.17)	2.52(1.11)	2.64(0.96)	2.50(0.77)	2.64(0.99)
Social self-care (scale 1–5)	3.98(0.64)	3.71(0.66)	4.03(0.53)	3.84(0.75)	3.92(0.65)	4.00(0.86)	3.93(0.68)	3.93(0.67)	3.87(0.71)	4.02(0.54)
Over-identification (scale 1–5)	3.08(0.97)	2.67(3.19)	2.92(0.95)	3.11(1.09)	3.01(1.00)	3.13(1.49)	2.96(1.11)	3.01(1.01)	2.98(1.07)	3.06(1.03)
Self-kindness (scale 1–5)	3.25(0.82)	3.19(0.94)	3.34(0.89)	3.16(0.75)	3.23(0.83)	3.13(0.85)	3.37(0.98)	3.28(0.84)	3.37(0.86)	3.06(0.82)
Mindfulness (scale 1–5)	3.53(0.86)	3.84(0.78)	3.66(0.86)	3.51(0.84)	3.57(0.84)	3.50(0.71)	3.74(0.98)	3.58(0.88)	3.85(0.72)	3.40(9.82)
Isolation (scale 1–5)	2.77(1.00)	2.36(1.04)	2.56(0.92)	3.88(1.11)	2.74(1.02)	3.13(1.31)	2.30(1.00)	2.73(1.06)	2.69(1.05)	2.64(0.88)
Common humanity (scale 1–5)	3.22(0.85)	3.23(0.79)	3.31(0.91)	3.14(0.76)	3.20(0.85)	3.25(0.87)	3.35(0.90)	3.29(0.78)	3.20(1.09)	3.04(0.82)
Self-judgement (scale 1–5)	2.88(0.95)	2.51(1.23)	2.69(1.00)	3.97(1.01)	2.80(0.99)	2.13(1.31)	2.87(1.04)	2.85(1.04)	2.52(0.89)	2.94(0.97)
Compassion satisfaction (scale 0–15)	12.09(2.78)	11.68(3.04)	12.38(2.33)	11.66(3.28)	11.92(2.80)	13.00(2.71)	12.09(3.12)	11.94(2.86)	12.79(2.32)	11.69(3.04)
Compassion fatigue (scale 0–15)	3.55(2.94)	3.21(3.06)	3.15(2.68)	3.89(3.24)	3.66(2.91)	3.00(3.56)	2.96(3.21)	3.59(3.07)	3.32(2.60)	3.31(2.91)
Burnout (scale 0–15)	6.61(3.70)	5.56(4.21)	5.90(3.46)	7.03(4.12)	6.62(3.69)	5.25(4.57)	5.70(4.15)	6.85(3.79)	4.75(3.00)	6.51(4.10)
Life satisfaction—item 1 (scale 1–5)	2.87(1.12)	3.04(1.23)	3.13(1.13)	2.67(1.10)	2.89(1.19)	2.75(0.96)	3.04(0.98)	2.92(1.19)	3.21(1.03)	2.60(1.04)
Life satisfaction—item 2 (scale 1–5)	3.54(0.99)	3.54(1.11)	3.69(0.92)	3.38(1.10)	3.53(1.03)	3.25(0.50)	3.57(1.04)	3.54(1.02)	3.68(1.02)	3.43(1.01)
Life satisfaction—item 3 (scale 1–5)	3.10(0.93)	3.11(1.23)	3.29(0.86)	2.89(1.06)	3.07(0.99)	3.00(0.82)	3.30(1.02)	3.14(0.96)	3.14(1.08)	2.94(0.95)
Life satisfaction—item 4 (scale 1–5)	3.06(0.97)	3.32(1.16)	3.27(0.98)	2.94(0.99)	3.09(1.02)	2.50(0.58)	3.30(0.97)	3.16(1.01)	3.21(1.10)	2.86(0.91)
Life satisfaction—item 5 (scale 1–5)	3.90(1.03)	3.82(1.19)	4.03(0.99)	3.72(1.12)	3.82(1.10)	3.50(0.58)	4.17(0.83)	3.94(1.03)	3.96(1.14)	3.63(1.06)
Life satisfaction (scale 1–5)	3.29(0.87)	3.36(1.04)	3.48(0.85)	3.12(0.91)	3.29(0.93)	3.00(0.37)	3.48(0.80)	3.34(0.90)	3.44(0.92)	3.08(0.84)

Notes: Mean values outside parentheses; standard deviation values inside parentheses.
